# Supplementary material for: Complex‐centric proteome profiling by SEC‐SWATH‐MS
Source: Mol Syst Biol. 2019 Jan 14;15(1):e8438. doi: 10.15252/msb.20188438 (PMC6346213; doi:10.15252/msb.20188438)
Supplement: Supplementary file 8 — Dataset EV7 [file MSB-15-e8438-s008.zip › feature_plots_string/Q6P1L8.pdf]

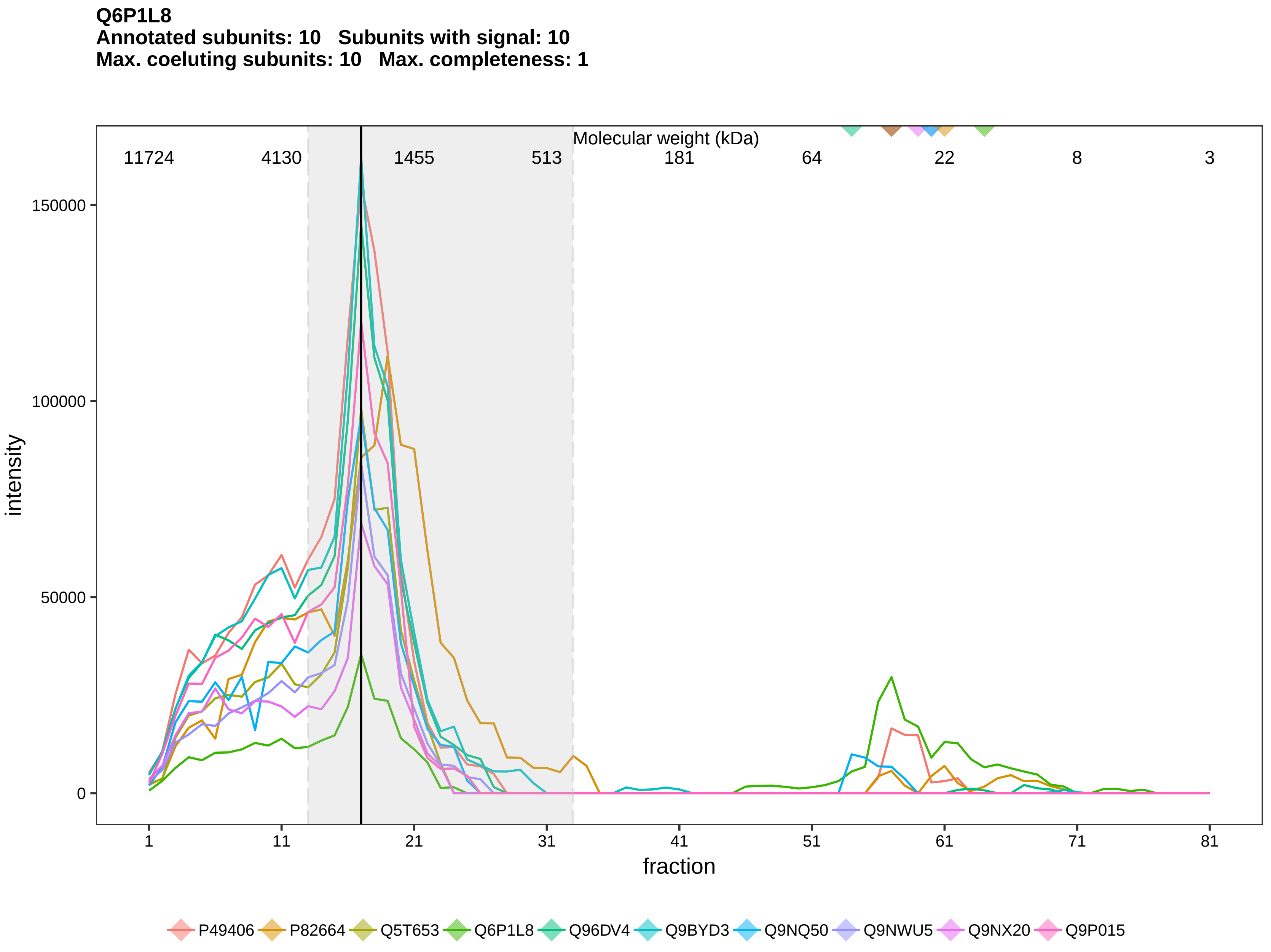
Q6P1L8
Annotated subunits: 10 Subunits with signal: 10
Max. coeluting subunits: 10 Max. completeness: 1
Molecular weight (kDa)
11724
4130
1455
513
181
64
22
8
3
intensity
150000
100000
50000
0
fraction
1
11
21
31
41
51
61
71
81
P49406
P82664
Q5T653
Q6P1L8
Q96DV4
Q9BYD3
Q9NQ50
Q9NWU5
Q9NX20
Q9P015
